# Modulating Th17-driven inflammation in ulcerative colitis with Chinese medicine: pathways, cytokines and beyond

**DOI:** 10.1186/s13020-026-01499-6

**Published:** 2026-10-29

**Authors:** Yushi Tian, Yuyi Yuan, Zhiqiang Zhao, Jingjing Li, Gengchen Xue, Jingbo Wang, Shuangjiao Deng, Heng Fan, Yujin Liu

**Affiliations:** Department of Integrated Traditional Chinese and Western Medicine, Union Hospital, Tongji Medical College, Huazhong University of Science and Technology, Wuhan, 430022 China

**Keywords:** Traditional Chinese medicine, Th17 cells, Cytokines, Ulcerative colitis, Colitis-associated biological processes

## Abstract

Ulcerative colitis (UC) is a type of chronic non-specific inflammation of the digestive tract, whose incidence is increasing gradually. Th17 cells and their cytokines play a critical role in the inflammatory response of UC. Th17 cells need a particular environment for the production of cytokines and exert their immunoregulatory functions. They promote inflammation and tissue healing and interact with other types of immune cells. The abundance of Th17 cells and their cytokine expression capability were found to significantly change in the intestines of patients with UC. In this paper, we discussed how traditional Chinese medicines (TCMs) regulate the function of Th17 cells and cytokine expression in UC. Chinese medicine monomers (e.g., matrine, paeoniflorin, curcumin, etc.) and compound decoctions (e.g., compound Sophorae decoction, Gegen Qinlian decoction, QingChang-XiaoPi decoction, etc.) were shown to regulate the behavior of Th17 cells and their cytokine capability by modulating several signaling pathways. These TCMs inhibited cytokine secretion, improved the intestinal bacterial flora, restored Th17/Treg cell balance, and alleviated intestinal inflammation. TCM offers a wide variety of molecules that modulate the Th17 response and the development of UC. The use of these molecules, in conjunction with conventional treatments, may provide a better therapeutic option for UC.

**Graphical Abstract:**

**Figure:**
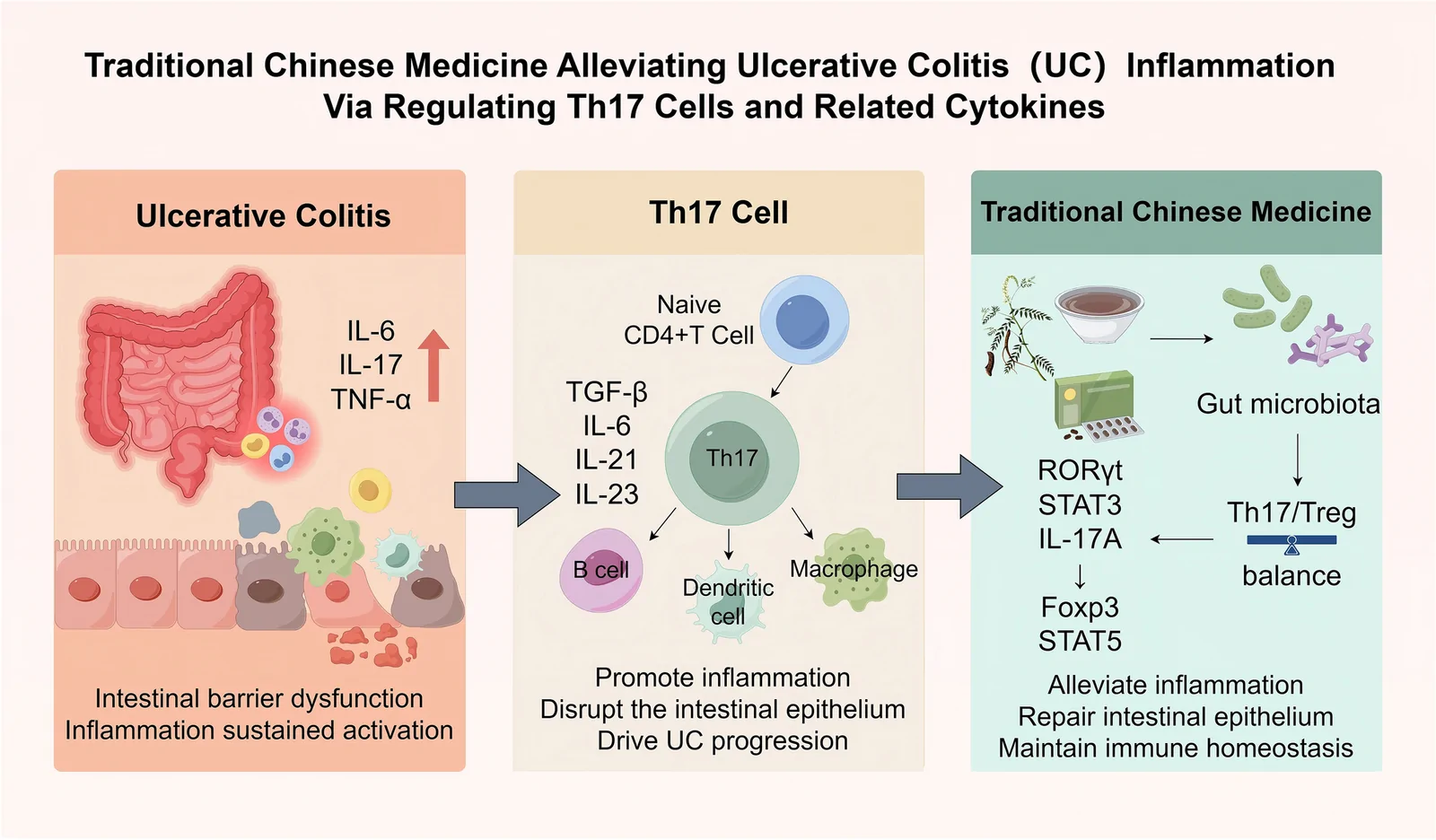

## Introduction

UC is a chronic non-specific inflammatory disease of the intestinal tract characterized by persistent inflammation of the colorectal mucosa. The typical symptoms of UC include diarrhea, mucopurulent bloody stools, abdominal pain, and urgency. These symptoms persist in the long term and manifest in cyclic episodes, seriously affecting the quality of life of patients. In recent years, with changes in dietary structure, environmental pollution, and increased mental stress, the incidence of UC has significantly increased globally, imposing a heavy burden on society [1]. Europe and North America exhibit the highest incidence and prevalence of UC, with the prevalence of UC exceeding 0.3% of the total population in many countries [2, 3]. In Japan, the annual incidence rate per 100,000 population has risen dramatically from 5 in 2010 to 98 in 2019 [4]. Similarly, the annual incidence rate has also been growing rapidly in other Asian countries. For instance, it is estimated that the number of people suffering from UC in China has exceeded 1 million [5], imposing a huge burden on healthcare resources. UC not only increases medical expenditures but also undermines the function and quality of life of patients, making it a major challenge in the field of public health.

The mechanisms underlying UC remain largely unclarified, yet extensive research indicates that impaired immune function plays a pivotal role in the development of UC [6]. Within this context, Th17 cells, representing a significant subset of CD4+ T-cells, possess a vital function in gut immunity and inflammatory responses [7]. Th17 populations are generated and matured in the presence of particular cytokines, such as TGF-β, IL-6, IL-21, and IL-23. After activation, Th17 releases massive amounts of pro-inflammatory molecules, including IL-17, IL-6, and TNF-α, inducing epithelial cells, fibroblasts, and macrophages to synthesize high levels of inflammatory factors, thereby facilitating the accumulation of immune cells, like neutrophils and monocytes, thereby intensifying the primary injury and intestinal inflammation [8, 9]. Specifically, IL-17 can weaken mucosal defense mechanisms, facilitating the mucosal penetration of harmful bacteria and toxins [10]. Furthermore, Th17 cells can engage with diverse immune elements, including B cells and dendritic cells, thereby affecting the immunological equilibrium in the gut mucosa [11]. Consequently, Th17 cells are regarded as a primary cell subset driving the persistent inflammatory state in UC, and their activation pathways and pathogenic profiles are becoming emerging targets for the management of inflammatory bowel disease. Beyond T-cell development and multiplication, the migration of T-cell groups is a fundamental factor in mucosal defense. The orphan G protein-coupled receptor GPR15 has recently emerged as a key chemotactic receptor mediating the homing of regulatory and effector CD4⁺ T cells to the colonic lamina propria, acting in concert with its endogenous ligand (GPR15L) encoded by the C10orf99 gene [12, 13]. GPR15 and GPR15L form a heterodimer that is required to recruit lymphocytes to particular areas in the intestine since they transmit additional inhibitory or stimulatory signals. GPR15 expression is also regulated by aryl hydrocarbon receptor (AHR) signaling. In fact, AHR-responsive dietary or microbial metabolites can modify GPR15 expression on CD4⁺ T cells to connect environmental and microbial signals to T-cell migration in the intestines [14, 15].

TCM is a system of multiple targets and can treat various manifestations of UC. TCM can simultaneously modulate different biological pathways, regulate inflammation, modify the composition of gut microbiota, strengthen the intestinal mucosal barrier, and maintain immune homeostasis [16–18]. Most of these effects are mediated by alterations in key immune pathways regulating Th17 cell differentiation and function. TCM monomers, such as paeoniflorin, matrine, baicalein, and curcumin, were shown to modulate key signaling pathways involved in the activation and maintenance of Th17 cells, including the JAK/STAT pathway, NF-κB, and AhR/IL-22 pathways. These pathways regulate the differentiation of Th17 cells, promote the secretion of pro-inflammatory cytokines, and maintain immune homeostasis [19, 20]. Compound Sophorae decoction and Gegen Qinlian decoction can also be used in combination to regulate Th17 behavior, alleviate gut dysbiosis, and improve the mucosal barrier function. These formulas can also alter the differentiation of Th17 cells and inhibit immune-modulating pathways, such as the JAK/STAT, MAPK, and mTOR pathways, to correct immune dysregulation and inflammation. Together, they form a holistic approach to the treatment of UC [21, 22].

By searching PubMed, we identified studies on how TCMs affect Th17 cells in UC. Studies published from January 2010 to December 2025 were collected. The keywords included "ulcerative colitis", "Th17", "regulatory T cells", "traditional Chinese medicine", "herbal compounds", and "decoction/formula". Only studies published in English were included. We focused on studies investigating the effects of single TCM compounds or compound formulas on Th17 cells, Th17/Treg balance, cytokine production, and intestinal inflammation in UC. In vitro studies, animal experiments, and clinical trials were included, while review articles, commentaries, and studies that did not focus on Th17 modulation or UC were excluded. Therefore, we unraveled the mechanism of action of TCMs in UC based on preclinical data and delineated deficiencies in clinical research.

The central framework of this review is as follows: TCM intervention → modulation of Th17 cell behavior and associated immune pathways → amelioration of UC-related inflammation, immune imbalance, and mucosal injury. This review systematically explores how TCM monomers and formulas regulate Th17-related pathways and discusses their potential applications in the treatment of UC.

## The biological properties of Th17 cells

### Activation characteristics

**Fig. 1:**
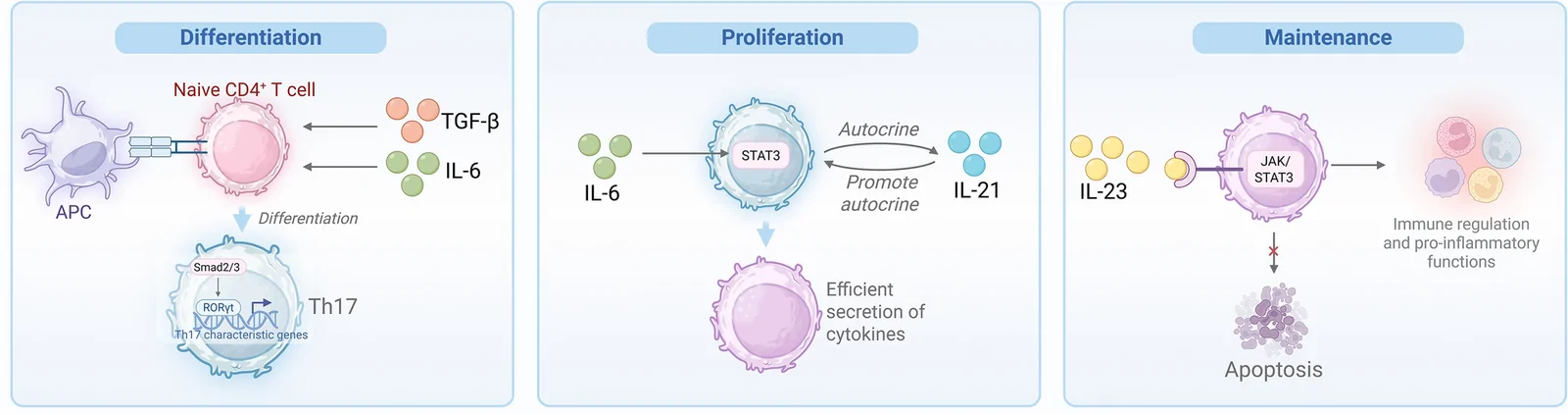
The activation process of Th17 cells

Th17 cells are a subpopulation of effector helper T-cells formed via the differentiation of naive CD4^+^T cells in the presence of specific inflammatory factors. Th17 cell activation is a complex and fine-tuned process that requires specific cytokines for its initiation and maintenance. This process mainly involves differentiation, expansion, proliferation, and functional maturation and maintenance [23, 24]. Cytokines such as TGF-β, IL-6, IL-21, and IL-23 play key roles in Th17 cell activation. TGF-β plays a critical role in guiding the initial stage of Th17 cell activation. TGF-β provides basic signals for the differentiation of naive T cells to Th17 cells, prompting T cells to embark on the path of transformation to Th17 cells. IL-6, on the other hand, activates a series of signaling pathways to induce the expression of specific transcription factors by T cells, laying the foundation for the expression of characteristic genes in mature and activated Th17 cells. The synergistic effect of IL-6 and TGF-β can strengthen this differentiation process [25]. Meanwhile, co-stimulation of TGF-β and IL-6 activates STAT3, resulting in the production of RORγt, a key transcription factor for Th17 differentiation. IL-6 stimulates the production of IL-21 via STAT3 [26]. IL-21 plays an important autocrine regulatory role in Th17 cell activation, and enhances the metabolic activity and function of Th17 cells by activating relevant signaling pathways, enabling them to more efficiently secrete cytokines and amplify the immune effects of Th17 cells [27]. IL-23 plays a key role in maintaining and stabilizing Th17 cell activation [28]. After binding to its receptor on the surface of Th17 cells, IL-23 activates subsequent signaling pathways. This keeps Th17 cells alive and in an active state for a long time. IL-23 also inhibits Th17 cell apoptosis and maintains their immunomodulatory and pro-inflammatory functions (Fig. 1) [29, 30].

### Functional properties

#### Immunomodulation

Th17 cells play a multidimensional immunoregulatory role in the mucosal immune system, and their functions encompass inflammatory cell recruitment, immune cell activation, cytokine regulation, and immune network integration. As one of the major effector subpopulations of T-cells, IL-17 secreted by Th17 cells can significantly stimulate epithelial cells, fibroblasts, and immune cells to produce a large amount of chemokines (e.g., CXCL1, CXCL2, CXCL8) and granulocyte colony-stimulating factor (G-CSF). These chemokines and G-CSF can rapidly mobilize neutrophils to the site of inflammation or infection, thereby playing a pivotal role in the first line of defense [31, 32]. Meanwhile, IL-17 enhances the production of pro-inflammatory factors, such as IL-6, IL-1β, and TNF-α, by dendritic cells and macrophages, forming an inflammatory cascade and expanding the breadth and persistence of the immune response [33].

Th17 cells also form a synergistic network with other immune cells to induce a coordinated immune response. On the one hand, IL-21 secreted by Th17 cells serves as an important autocrine factor for self-expansion, which stabilizes the Th17 phenotype and enhances cytokine secretion by Th17 cells [34]. On the other hand, IL-21 promotes B-cells to differentiate into plasma cells, enhances antibody production, and strengthens the humoral immunity [35]. In addition, Th17 cells can activate or enhance the maturation and antigen presentation function of dendritic cells, thereby increasing the activation efficiency of newly formed T cells. The cytokines secreted by Th17 cells can also affect CD8^+^T cell function, forming a more complex immune-linked mechanism [36].

There is also a close antagonistic relationship between Th17 cells and regulatory T cells (Tregs). Initially, TGF-β signaling regulates the development of both cell types during their differentiation. When there are more inflammatory mediators in the environment, like IL-6, IL-21, and IL-23, the differentiation bias favors Th17 cells. This boosts RORγt activity, downregulates Foxp3 expression, and impairs the balance between Th17 and Treg cells in the immune system, making the immune system less tolerant and more proinflammatory [37]. This mechanism is considered a primary pathogenic basis for several chronic inflammatory conditions, including UC [38].

Th17 cells exhibit phenotypic plasticity. When exposed to inflammation for an extended period, Th17 cells may produce IFN-γ. IFN-γ transforms Th17 cells into Th1-like IL-17^+^IFN-γ^+^ double-positive cells, thereby intensifying the inflammatory response [39]. This phenotypic change shows that Th17 cells have many functions in different areas of the immune microenvironment and are likely to help maintain chronic inflammation and contribute to the progression of autoimmune diseases.

#### Effects that lead to inflammation

Th17 cells play key roles in many autoimmune and chronic inflammatory diseases. They release IL-17A and IL-17F, which are essential for the initiation and propagation of inflammation. These cytokines help immune cells reach the affected area, start an inflammatory response, and maintain this response.

Th17 cells produce a great proportion of IL-17A [40]. It binds to IL-17 receptor on the surface of other cells and induces the release of inflammatory molecules, like prostaglandin E2 and nitric oxide. These molecules worsen the inflammation by bringing more immune cells to the area [41]. IL-17A also activates several pathways, like NF-κB, MAPK, and C/EBP, potentiating the production of other pro-inflammatory cytokines, such as IL-6, IL-1β, TNF-α, G-CSF, and various chemokines. Therefore, more immune cells enter the area, which worsens the inflammation [42–44]. IL-17A also upregulates the production of matrix metalloproteinases, such as MMP-1, MMP-3, and MMP-9. These enzymes break down the extracellular matrix and basal membranes, damaging the tissue and promoting inflammatory cell infiltration [45]. IL-17F also plays a key role in the pro-inflammatory effects of Th17 cells. It is similar to IL-17A, with 50% similarity in its amino acid sequence. Both of them are secreted by Th17 cells. IL-17F can form homodimers (IL-17F/IL-17F) or heterodimers (IL-17A/IL-17F). These dimers bind to the IL-17RA/IL-17RC receptor complex on target cells, initiating the signaling process [46, 47]. IL-17F activates pathways like NF-κB, MAPK (p38, ERK1/2), and ACT1–TRAF6. Therefore, the target cells are more likely to produce pro-inflammatory cytokines, chemokines, and antimicrobial peptides, thereby sustaining the inflammatory response [48–51]. However, IL-17F exhibits a weaker effect than IL-17A. It is mainly active in the chronic stage of inflammation or under mild stimulation; therefore, it is less cytotoxic. It can help keep the balance of inflammation and regulate signals in the mucosal immune system [52].

Th17 cells can exhibit stronger inflammatory responses under certain inflammatory conditions and transform into pathogenic pTh17 cells [53]. These cells are often identified in environments with elevated levels of IL-23 and IL-1β. They produce both IL-17A and IFN-γ and represent a mixed Th17/Th1 phenotype. They emit stronger inflammatory signals that can directly cause death and necrosis of the mucosal epithelial cells [54]. Studies have shown that pTh17 cells can increase their own activity and attract more immune cells by inducing key genes, such as IL-23R and RORC. Therefore, they induce uncontrolled tissue inflammation and epithelial cell death and promote disease progression [55, 56].

#### Role in tissue repair

During immune balance or after tissue damage, Th17 cells can promote inflammation and contribute to the reconstruction of the physiological barriers. This shows that they possess an "off-switch" mechanism for immunity [57]. IL-22 is a key repair-associated cytokine secreted by Th17 cells, which primarily acts on epithelial cells in the intestinal and skin barrier [58]. IL-22 activates the STAT3 signaling pathway to promote the proliferation and differentiation of epithelial cells. It can help maintain the normal function of tight junction proteins Claudin and Occludin to strengthen the mucosal barrier [59]. It also stimulates epithelial cells to produce antimicrobial peptides, such as RegIIIγ and β-defensin, thereby preventing pathogen proliferation, maintaining a stable microbial environment, promoting tissue repair, and strengthening the immune response [60].

In addition to IL-22, Th17 cells can produce other repair-related factors, like IL-26, GM-CSF, and amphiregulin (AREG) in certain environments. IL-26 is a member of the IL-10 family, found only in humans. It potentiates the antimicrobial barrier in epithelial cells and promotes mucosal tissue repair, especially in the respiratory and intestinal tract [61]. GM-CSF is another key factor secreted by Th17 cells. In inflammatory conditions, it contributes to the growth and activation of myeloid cells, boosting the immune response [62]. GM-CSF keeps tissue balance and facilitates extracellular matrix repair and regrowth in low-inflammation states [63]. Some Th17 subgroups produce AREG, which functions as an EGFR ligand. AREG directly stimulates epithelial cell growth and repair barrier tissues [64].

**Fig. 2:**
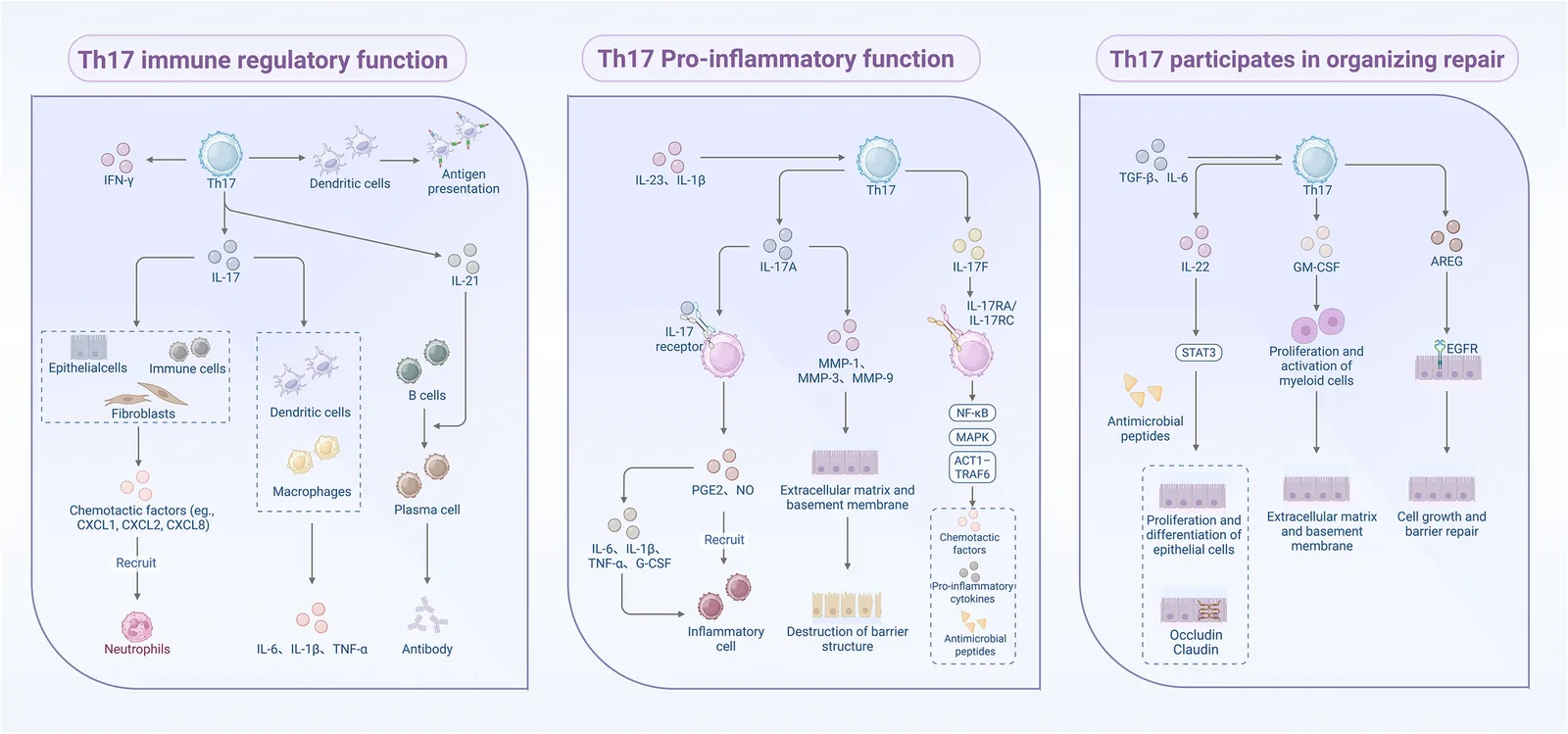
Functional characteristics of Th17 cells

The tissue repair function of Th17 cells mainly originates from the non-pathogenic subtype (non-pTh17). These cells typically differentiate in the presence of TGF-β alone or the combination of TGF-β and low concentrations of IL-6. They primarily secrete cytokines, like IL-22 and IL-10, produce less IL-17A and IFN-γ, and exhibit weaker tissue toxicity [65]. Under steady-state conditions, non-pTh17 cells are widely distributed in the lamina propria of various mucosal tissues. They help maintain barrier integrity, prevent microbial invasion, rapidly respond to injury, and accelerate tissue repair [66]. Compared to pro-inflammatory pTh17 cells, non-pTh17 cells promote a "protective" immune response, forming an important immune barrier that maintains tissue homeostasis (Fig. 2).

### Additional regulatory dimensions of Th17 cells

#### Metabolic reprogramming of Th17 cells

Th17-cell differentiation and effector function are not controlled only by cytokine signaling and lineage-defining transcription factors. They are also closely linked to metabolic reprogramming. After activation, CD4⁺ T cells undergo clear metabolic changes to support cell growth, proliferation, and functional differentiation [67]. Compared with Treg cells, Th17 cells rely more on glycolysis, changes in amino acid consumption pattern, and lipid metabolic remodeling [68, 69]. These metabolic pathways provide energy and biosynthetic materials for cytokine production and sustained inflammation. Recent studies have shown that pathogenic Th17 cells need specific bioenergetic programs to maintain their effector phenotype. One study found that STAT3 S727 phosphorylation maintains both glycolytic capacity and pathogenic effector identity in Th17 cells. Disruption of this signaling destabilizes the pathogenic program of Th17 cells [70]. Lipid metabolism is also critical to this process. A recent study showed that de novo sphingolipid biosynthesis is needed for metabolic adaptation during Th17 differentiation. It also helps maintain the function of effector Th17 cells. Enzymes involved in sphingolipid synthesis, such as SPTLC1, are linked to ROS balance and metabolic fitness in Th17 cells [71]. Glycolytic control in Th17 cells is not always proinflammatory. Phosphoglycerate mutase (PGAM), which catalyzes the conversion of 3-phosphoglycerate to 2-phosphoglycerate, was shown to limit the pathogenicity of Th17 cells by suppressing serine-dependent inflammatory epigenetic reprogramming. PGAM inhibition upregulated the expression of pathogenic genes and worsened Th17-driven autoimmunity in previous studies [72].

#### Epigenetic regulation and Th17 cell plasticity

Th17 cells are regulated not only by transcription factors, such as RORγt and STAT3, but also by epigenetic mechanisms. These mechanisms shape the identity and functional diversity of Th17 cells. Recent studies have indicated that pathogenic and non-pathogenic Th17 cells are linked to different chromatin programs [73]. BACH2 represents a crucial regulator of this process. A recent study showed that BACH2 promotes immunomodulatory non-pathogenic Th17 programs and suppresses proinflammatory Th1-like Th17 states. This finding suggests that changes in chromatin state can affect the regulatory features or inflammatory activity of Th17 cells [74]. Chromatin accessibility is also involved in the pathogenic function of Th17 cells. Another study found that pathogenic Th17 cells have specific proximal and distal chromatin-accessible regions. These areas are connected to the primary disease-related paths, and it can be concluded that chromatin accessibility supports the pathogenic state of Th17 cells [75]. Altered RNA expression can also act as a functional regulator. Studies on pathogenic CD4⁺ T-cells have shown that the m6A demethylase ALKBH5 promotes inflammatory immune programs and supports autoimmune responses. This finding suggests that RNA methylation may also contribute to Th17-associated inflammatory activity [76].

#### Intestinal microenvironment and Th17 cell regulation

Th17 cells are not only regulated by intracellular signaling pathways but also affected by the intestinal microenvironment. In the gut, Th17 cell responses are modulated by epithelial cells, antigen-presenting cells, regulatory lymphocytes, resident microbiota, microbial metabolites, and local inflammatory mediators. These local factors determine whether Th17 cells develop protective functions or pathogenic functions [77]. Local regulatory networks play major roles in this process. Intestinal Treg cells can selectively limit mucosal Th17 responses through IL-27-dependent mechanisms. Impairment of this regulatory pathway enhances intestinal Th17 responses and worsens intestinal inflammation. This shows that gut-specific control of Th17 immunity depends not only on intrinsic T-cell pathways but also on local immune regulation [78]. Microbial metabolism is another important factor in this process. Experimental studies have shown that the gut bacterium *Eggerthella lenta* can directly promote intestinal Th17 activation and worsen colitis in a strain-dependent manner. Bacterial metabolic activity promotes the function of the Th17 transcription factor RORγt. These results suggest that microbial metabolism can actively promote Th17-mediated intestinal inflammation and is not restricted to a background state [79].

Modified tryptophan metabolism is now known to be associated with altered immune states in diseased intestines. Recently, scholars have discovered that the breakdown of tryptophan in the gut is linked to the immune function of the gut. Therefore, it can be assumed that changes in metabolite pathways are responsible for mucosal immune dysregulation and prolonged inflammation [80]. In addition to controlling the differentiation of Th17 and Treg cells, gut microbiota-derived and diet-related metabolites can regulate T cell homing receptors via AHR. Recently, studies have shown that food factors, such as L-tryptophan, can adjust the activity of AHR and change the amount of GPR15 on CD4⁺ T cells that migrate to the colonic mucosa and modulate the severity of colitis [81–83]. AHR integrates signals from host and microbial metabolites to regulate the immune gene network that includes chemotactic receptors, thereby linking environmental cues and microbial metabolism to T cell localization and mucosal immune homeostasis [84]. TCM can alter the composition of the gut microbiome and its metabolism, thereby promoting AHR-mediated upregulation of GPR15 and reshaping the spatial distribution of regulatory and effector T-cells in UC.

## Relationship between Th17 cells and related cytokines in UC

Both the abundance and functions of Th17 cells, as well as their cytokines, were found to change in the intestines of patients with UC. Studies have shown that the number of Th17 cells is increased in the intestinal mucosa of patients with UC, accompanied by a significant increase in the expression levels of cytokines, such as IL-17 and IL-22. IL-17 is a typical interleukin that contributes to the development and progression of UC. At the site of damage in the intestinal lining, it mobilizes inflammatory cells, such as neutrophils and monocytes [85]. These cells also secrete other damaging factors, including prostaglandin E2 and nitric oxide, which further exacerbate inflammation [86]. Moreover, IL-17 can damage the intestinal barrier and increase its permeability, thus allowing bacteria and other toxins to penetrate the underlying tissues and intensify the severity of UC [87]. Generally, IL-17 levels are relatively high in individuals with UC and are positively correlated with the degree of inflammation [88]. In addition, IL-17 drives intestinal fibrosis in UC. Fibrosis can damage the tissue, reduce its function, and contribute to intestinal narrowing [89]. IL-17 stimulates fibroblasts to produce fibrosis-related molecules, such as collagen types I and III, TGF-β1, and α-SMA, thereby increasing tissue stiffness and matrix accumulation. Anti-IL-17 agents were shown to prevent colonic fibrosis in mice with UC by downregulating collagen type III and pro-fibrotic cytokines [90]. Recent studies have shown that IL-17 can directly damage neurons and glial cells in the intestine. It downregulates the release of neurotransmitters and enhances intestinal sensitivity; thus, patients with UC may experience more severe abdominal pain and frequent urges to defecate [91, 92]. Under chronic inflammation, Th17 cells experience mitochondrial stress, leading to mitochondrial dysfunction and the accumulation of reactive oxygen species (ROS) [93]. Patients with UC have significantly higher levels of IL-22. IL-22 can promote the proliferation, migration, and differentiation of intestinal cells to enhance the integrity of the intestinal barrier and improve mucosal function. It can also stimulate epithelial cells to produce antimicrobial peptides, such as β-defensins and RegIIIγ, strengthen local defense against pathogens, and prevent pathogen invasion. Moreover, it induces the development and maturation of goblet cells, promotes mucin secretion (particularly MUC2 secretion), and repairs the damaged mucosal layer in patients with UC [94, 95]. However, overly high levels of IL-22 may induce abnormal cell proliferation and pathological inflammation in prolonged inflammatory diseases; therefore, its effect on UC relies on the microenvironment [96]. IL-17 and IL-23 also play key roles in the maintenance of Th17 cell function in UC. IL-23 is a central regulator of the inflammatory response in UC. Upon stimulation with pathogen-related receptors, such as TLRs, dendrites in the intestine of patients with UC release a large amount of IL-23. Compared to the mucosa of healthy patients, IL-23 is significantly overexpressed in the mucosa of patients with UC [97]. It binds to IL-23R on Th17 cells and activates transcription factors, such as STAT3 and BATF, thereby maintaining the activity of Th17 cells. IL-23 also inhibits Th17 cell-mediated production of anti-inflammatory factors, such as IL-10. Clinical data showed that patients with UC have higher serum levels of IL-23 compared to healthy individuals. Elevated levels of IL-23 were found to be closely associated with the severity of intestinal mucosal injury, C-reactive protein (CRP) levels, and colonoscopy scores; thus, IL-23 may be a cause of continuous inflammation and mucosal damage in UC [98].

**Fig. 3:**
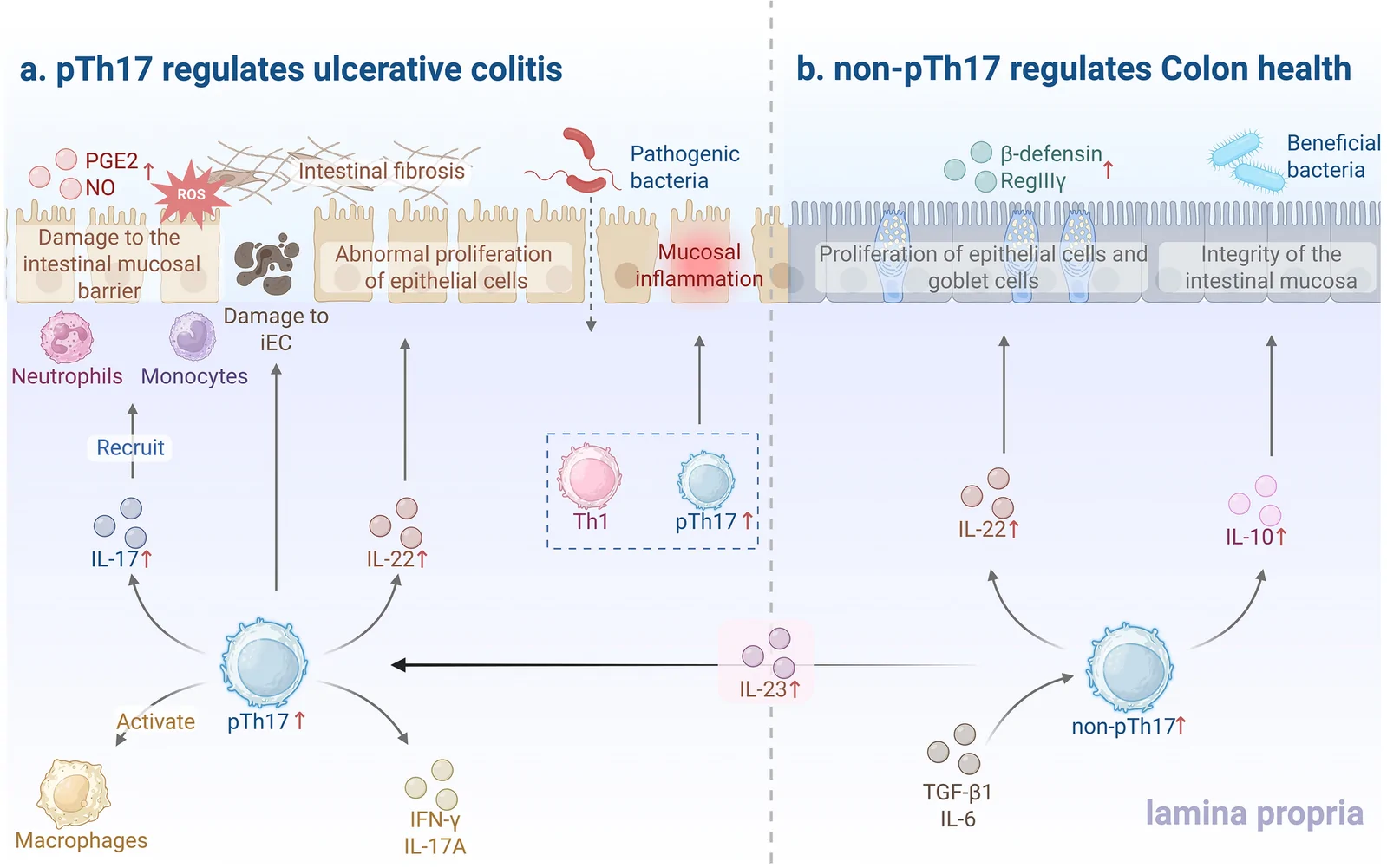
The role of Th17 cells and cytokines in the development and progression of UC

With the deepening of research on the role of Th17 cells in UC, Th17 cells are deemed to damage the intestinal mucosa instead of maintaining intestinal balance. Th17 cells exhibit phenotypic heterogeneity, encompassing both immunosuppressive and proinflammatory responses. Induction experiments revealed that pathogenic Th17 (pTh17) cells co-express interferon-γ and IL-17A, while non-pathogenic Th17 (non-pTh17) cells, another type of Th17 cells, do not cause disease [99]. Under normal conditions, non-pTh17 cells are produced subsequent to *Clostridium difficile* exposure and reside in the intestinal lamina propria. These non-pTh17 release IL-10 to help maintain intestinal mucosal integrity and immune function [100]. In UC, TGF-β1 and IL-6 activate non-pTh17 cells, showing protective effects on the intestinal mucosa [101]. pTh17 cells induce intestinal epithelial cell damage by promoting the expression of UC susceptibility genes in these cells and triggering cellular death mechanisms [102]. Evidence from patients with IBD and animal models indicates that Th1 cells and pTh17 cells act synergistically during disease progression and exacerbate mucosal inflammation [103]. Simultaneously, pTh17 cells interact with other immune cells, activating macrophages to release inflammatory mediators and exacerbate intestinal inflammation [104]. Studies have shown that IL-23 induces pTh17 cells to produce GM-CSF and IL-22, forming an "inflammation-barrier disruption" axis [105]. The metabolic factor eNAMPT is also upregulated in IL-23-induced pTh17 cells. It boosts IL-17A and IL-22 production, suggesting its role in regulating inflammation and metabolism [106]. IL-23 also increases mitochondrial activity in pTh17 cells, thereby maintaining their function in low oxygen conditions. IL-23 blocks molecules, like miR-146a, preventing the transformation of Th17 cells to Tregs or non-pTh17 [107, 108]. In UC, IL-23 is strongly co-expressed with Th17-specific factors, like RORγt and CCR6, supporting its role in the maintenance of the pTh17 lineage [109]. Recent studies have shown that pTh17 cells activate the HRas-STAT3 pathway. This pathway enhances their differentiation and lowers Foxp3 expression in Tregs, disrupting the Th17/Treg balance and intensifying inflammation. Th17/Treg imbalance leads to excessive Th17 activation and disrupts the intestinal immune homeostasis [110, 111]. Alterations in the composition of gut microbiota are critically involved in the differentiation of pTh17. Pathogenic bacteria, such as *Escherichia coli*, stimulate dendritic cells via TLR4 and AHR pathways, resulting in the production of IL-23 and IL-6 and expanding the pTh17 lineage [112, 113]. How pTh17 cells transform into non-pTh17 subtypes has become a major research focus [114]. In-depth research on the relationship between these two subtypes of Th17 and UC may provide novel targets and strategies for the diagnosis and treatment of UC (Fig. 3).

## Regulation of Th17 cells and their cytokines by TCM in the treatment of UC

Recently, research on the regulatory mechanisms of Th17 cells and their inflammatory cytokines has become a key focus in the treatment of UC using TCMs. TCMs can target many pathways, work holistically, and are safe, providing potential benefits in treating immune diseases. This section reviews available studies on how TCMs affect Th17 cells and their cytokines, focusing on TCM extracts and formulas.

### Regulation by TCM extracts

**Fig. 4:**
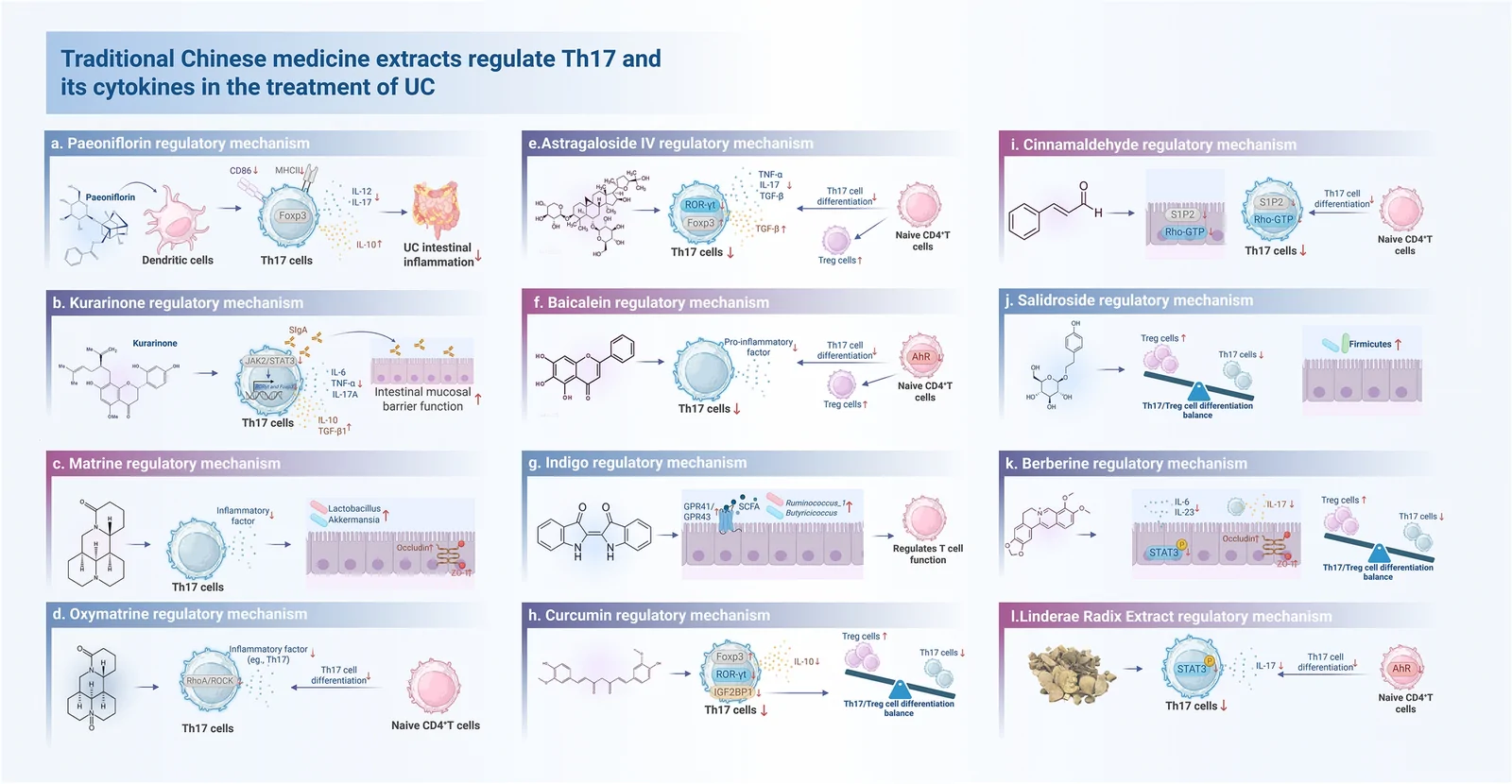
Interventional effect of TCM extracts on Th17 related mechanism in UC

Research shows that Th17 cells and their cytokines are important in UC. Many studies have looked at how TCM extracts can target these cells and cytokines to treat UC (Table 1, Fig. 4).

**Table 1:** Interventional effect of TCM extracts on Th17 related mechanism in UC

TCMs	Component types	Th17 related mechanism	Model	References
Paeonia lactiflora	Paeoniflorin	Reduction of IL-12 secretion in vitro and in vivo by regulating dendritic cells	TNBS Mice	[118]
Sophora flavescens	Kurarinone	downregulate IL-17A, and inhibit Th17 cell differentiation via JAK2/STAT3 pathway	DSS Mice	[122]
Sophora flavescens	Matrine	reduced the production of inflammatory cytokines amd adjusting the Th17/Treg ratio	DSS Mice	[123]
Sophora flavescens	Oxymatrine	downregulate IL-17A, and inhibit Th17 cell differentiation via RhoA/ROCK pathway	DSS Mice	[124]
Astragalus membranaceus	Astragaloside Ⅳ	Inhibit Th17 cell differentiation by downregulating ROR-γt and IL-17, and promote Treg-associated Foxp3 and TGF-β expression to restore immune balance	DSS Mice	[126]
Scutellaria baicalensis	Baicalein	Suppress Th17 cell differentiation and downregulate IL-6 and TNF-α while upregulating TGF-β and IL-10 expression via AhR activation	DSS Mice	[128]
Indigo naturalis	Indigo	Enhances SCFA–GPR41/43 signaling and increases butyrate production to indirectly suppress Th17 cell differentiation	DSS Rat	[131]
Curcuma longa	Curcumin	Regulates Th17/Treg cell balance through IGF2BP1-mediated m6A modification of LRP5	DSS Mice	[133]
cinnamon	Cinnamaldehyde	Inhibits Th17 cell differentiation through the S1P2 pathway and regulates lncRNA H19 and MIAT expression	DSS Mice	[135]
Rhodiola	Salidroside	Restores Th17/Treg balance by modulating gut microbiota composition, particularly through enrichment of Firmicutes	DSS Mice	[137]
Coptis chinensis Franch	Berberine	alleviates colonic inflammation by suppressing Th17 cell differentiation and IL-17 production, modulating gut microbiota composition to restore Th17/Treg balance, and enhancing Wnt/β-catenin signaling, collectively maintaining intestinal immune homeostasis in a microbiota-dependent manner	DSS Mice	[140–142]
Linderae Radix	Linderae Radix Extract	Inhibits Th17 cell differentiation by suppressing IL-6 secretion and blocking IL-6/STAT3 signaling pathway	DSS Mice	[144]

#### Paeoniflorin

Paeoniflorin, a derivative of Paeonia lactiflora root, possesses immune-modulating and anti-inflammatory properties [115, 116]. In a mouse model of TNBS-induced UC, treatment with paeoniflorin (50 mg/kg/day for 7 days) lowered DAI scores, reduced inflammation, and repaired mucosal damage [117]. Studies have shown that paeoniflorin modulates the function of Th17 cells by acting on dendritic cells. Injecting paeoniflorin-treated dendritic cells into mice with TNBS-induced UC suppressed CD86 and MHCII expression, reduced IL-12 and IL-17 secretion, and upregulated Foxp3 and IL-10 expression. Paeoniflorin indirectly restored Th17/Treg balance and alleviated intestinal inflammation in UC [118].

#### Kurarinone

Sophora flavescens consists of two main types of active compounds: quinolizidine alkaloids (mainly matrine and oxymatrine) and flavonoids (e.g., kurarinone, etc.). The alkaloids possess vigorous anti-inflammatory, antitumor, immune-regulating, and organ-protective effects. The flavonoids are known for their anti-inflammatory, antioxidant, and antibacterial properties [119–121]. Li et al. established mouse models of UC and RAW 264.7 cell models, which were subsequently treated with Sophora flavescens flavonoids. The results indicated that these flavonoids affect Th17 cell differentiation in peripheral blood and suppress the inflammatory response. Among the flavonoids, kurarinone exhibited the best effect in terms of lowering IL-6 and TNF-α levels. It also protected the intestinal mucosal barrier by upregulating SIgA secretion. Kurarinone downregulated IL-17A secretion, upregulated TGF-β1 and IL-10 expression, and altered RORγt and Foxp3 levels in the colon. Mechanistically, kurarinone blocked the JAK2/STAT3 signaling pathway, thereby regulating Th17 cell differentiation in UC [122].

#### Matrine

Studies have shown that matrine ameliorates DSS-induced UC. It downregulated inflammatory cytokines and suppressed the immune and inflammatory responses by adjusting the Th17/Treg ratio. Matrines also attenuated oxidative stress and increased the abundance of beneficial bacteria, like *Lactobacillus* and *Akkermansia*. Additionally, matrine repaired intestinal tissue damage, restored tight junction proteins, like ZO-1 and Occludin, and strengthened the intestinal mucosal barrier [123].

#### Oxymatrine

In a mouse model of DSS-induced UC, low, medium, and high doses of oxymatrine (25, 50, and 100 mg/kg, respectively) improved the symptoms of colitis. Similar to the ROCK inhibitor Y-27632, it blocked the RhoA/ROCK signaling pathway. It lowered Th17-related proinflammatory factors, like IL-17A, reduced Th17 cell differentiation, upregulated immune-suppressing factors, and restored the Th17/Treg imbalance [124].

#### Astragaloside IV

Astragaloside IV is a natural compound derived from Astragalus membranaceus. It was shown to effectively attenuate free radicals, oxidative stress, and inflammation [125] and reduce the abundance of Th17 cells, thereby mitigating DSS-induced UC. Mice were given two doses of astragaloside IV with 2% DSS. The results showed lower disease activity scores and milder colonic damage after treatment with astragaloside IV. Particularly, astragaloside IV reduced the abundance of Th17 cells in the blood, lowered IL-17 and TNF-α levels, and downregulated ROR-γt. It also increased the abundance of Treg cells and upregulated the expression of TGF-β and Foxp3. These findings suggest that astragaloside IV inhibited Th17 cell differentiation and proinflammatory cytokine release while promoting Treg-related immune regulatory pathways. The effectiveness in the high-dose astragaloside IV (100 mg/kg/day) group was similar to that in the positive control group and better than that in the low-dose group, suggesting a dose-dependent effect. Thus, early treatment with astragaloside IV may restore the Th17/Treg balance in mice with UC [126].

#### Baicalein

Scutellaria baicalensis can be classified into two types based on morphology and medicinal value: Scutellaria baicalensis root and Scutellaria baicalensis rhizome. The rhizome, the woody part of the mature root, contains higher levels of flavonoids and exhibits stronger medicinal efficacy. It contains compounds, like baicalin, baicalein, and wogonoside, which possess anti-inflammatory, antioxidant, and immune-regulating properties [127]. Baicalein stops Th17 cell development and downregulates inflammatory cytokines. It activates the AhR pathway to ameliorate DSS-induced colitis. DSS upregulated IL-17, IL-6, and TNF-α in mice, but these effects were reversed by baicalein (10, 20, and 40 mg/kg). Baicalein also activates AhR and promotes its nuclear translocation, thereby enhancing Treg cell differentiation. Collectively, in CD4 + T cells, baicalein was found to activate AhR [128].

#### Indigo

Indigo, a pigment from indigo powder, exerts anti-inflammatory, immune-regulating, and antitumor effects [129]. In UC models, indigo exhibited unique effects on immune metabolism [130]. In mouse models of DSS-induced UC, treatment with indigo blue suppressed colonic inflammation, reduced epithelial cell shedding, fixed crypt structure, lowered DAI scores, and restored colonic length. Unlike other treatments, indigo mainly targeted the metabolic system of the gut. Furthermore, indigo increased the activity of the SCFA signaling pathway by raising the expression of GPR41 and GPR43, thereby modulating T-cell function. Indigo also increased the abundance of beneficial intestinal bacteria, such as *Ruminococcus_1* and *Butyricicoccus*, suggesting that it helps control inflammation through a “microecology-metabolism-immunity” pathway [131].

#### Curcumin

Curcuma longa, a culinary and medicinal herb from India, yields curcumin, a constituent isolated from its rhizomes. Curcumin exhibits anticancer, antibacterial, anti-inflammatory, and anti-aging properties [132]. In a murine model of DSS-induced UC, curcumin-chitosan microspheres (CCM) decreased the proportion of Th17 cells. CCM enhanced the mRNA levels of Foxp3 while diminishing the mRNA levels of RORγt and IL-10. PCR array analysis indicated that the m6A-binding protein IGF2BP1 is involved in CCM-mediated regulation of the Th17/Treg equilibrium. IGF2BP1 overexpression inhibited the capacity of CCM to regulate the conversion of Th17 and Treg cells. IGF2BP1 targets LRP5 and regulates its m6A modification. Modifying IGF2BP1 or LRP5 reversed the effects of CCM on Th17/Treg cells [133].

#### Cinnamaldehyde

Cinnamaldehyde, the active compound of cinnamon, possesses anti-inflammatory and antibacterial properties [134]. It was shown to decelerate the progression of colitis by controlling proinflammatory cytokines and reducing the abundance of Th17 cells. Metabolomics studies showed that cinnamaldehyde affects the sphingolipid pathway, thereby lowering the protein levels of S1P2 and Rho-GTP in the colonic tissue. In preclinical models, cinnamaldehyde inhibited Th17 cell development and affected the S1P2 and Rho-GTP pathways. Blocking S1P2 with JTE-013 reversed the effects of cinnamaldehyde on Th17 cells. This suggests that cinnamaldehyde may reduce the abundance of Th17 cells through the S1P2 pathway and change lncRNA H19 and MIAT levels to mitigate UC [135].

#### Salidroside

Salidroside is an active compound of Rhodiola. It possesses strong antioxidant and immune-regulating properties [136]. Salidroside was reported to maintain Th17/Treg balance in UC by modulating the composition of gut microbiota. In mice with DSS-induced UC, salidroside improved gut microbiota diversity and increased the abundance of Firmicutes. This helped balance the Th17/Treg ratio. Antibiotics killing gut bacteria reduced the efficacy of salidroside. Transplanting salidroside-treated fecal microbiota restored its effects, suggesting that salidroside controls immune balance through the “microbiota-Th17” axis. Salidroside also protected the gut by changing macrophage pyroptosis, thereby suppressing inflammation and protecting the mucosal barrier. This shows its potential for treating UC and other immune disorders [137].

#### Berberine

Berberine is a natural chemical derived from the traditional Chinese herb Coptis chinensis Franch. It exerts anti-inflammatory, antimicrobial, antioxidant, and immune-regulating effects and improves metabolism [138, 139]. Studies have reported that berberine reduces inflammation in UC by regulating Th17 cell function. In mice with DSS-induced acute and chronic colitis, treatment with berberine increased body weight, lowered DAI scores, extended colon length, and reduced tissue damage. It also restored the expression of tight junction proteins, including ZO-1 and Occludin, and decreased expression levels of IL-17 and ROR-γt. Berberine reduced IL-6 and IL-23 levels and inhibited STAT3 activation in colonic tissues. It also reduced IL-17 production by CD4⁺ T cells in the spleen and mesenteric lymph nodes. Besides, berberine prevented the transformation of naive CD4⁺ T cells to Th17 cells. Berberine also affected the Th17/Treg balance by modulating the composition of gut microbiota. 16S rRNA analysis showed that berberine reduced gut bacteria diversity and changed the abundance of *Desulfovibrio, Eubacterium,* and *Bacteroides*. After administering antibiotics to remove gut microbiota, berberine lost its ability to regulate the Th17/Treg ratio. Fecal microbiota from berberine-treated mice restored its effects in microbiota-deficient mice, suggesting that berberine controls immune balance through the “microbiota-Th17” axis. In another study, berberine reduced the abundance of ILC1 and Th17 cells while increasing the abundance of Treg and ILC3 cells. This immune balance was controlled through the Wnt/β-catenin pathway, whose blockade weakened the beneficial effects of berberine [140–142].

#### Linderae radix extract

Linderae radix is the dried root of the Lindera plant from the Lauraceae family. It is a TCM with a long history of use. It promotes Qi, relieves pain, soothes the liver, and treats diarrhea and dysentery [143]. A study used linderae radix extract to treat UC and determine its effect on Th17 cells and the IL-6/STAT3 pathway. Linderaea radix extract reduced IL-6 release and inhibited STAT3 phosphorylation, thereby inhibiting Th17 cell differentiation. Consistently, linderae radix extract reduced the abundance of Th17 cells in the mesenteric lymph nodes of mice, relieved symptoms, decreased intestinal permeability, and repaired damaged tissues. Thus, linderae radix extract may suppress the Th17 immune response by regulating the IL-6/STAT3 pathway and suppressing inflammation in UC [144].

### Regulation of TCM formulation

**Fig. 5:**
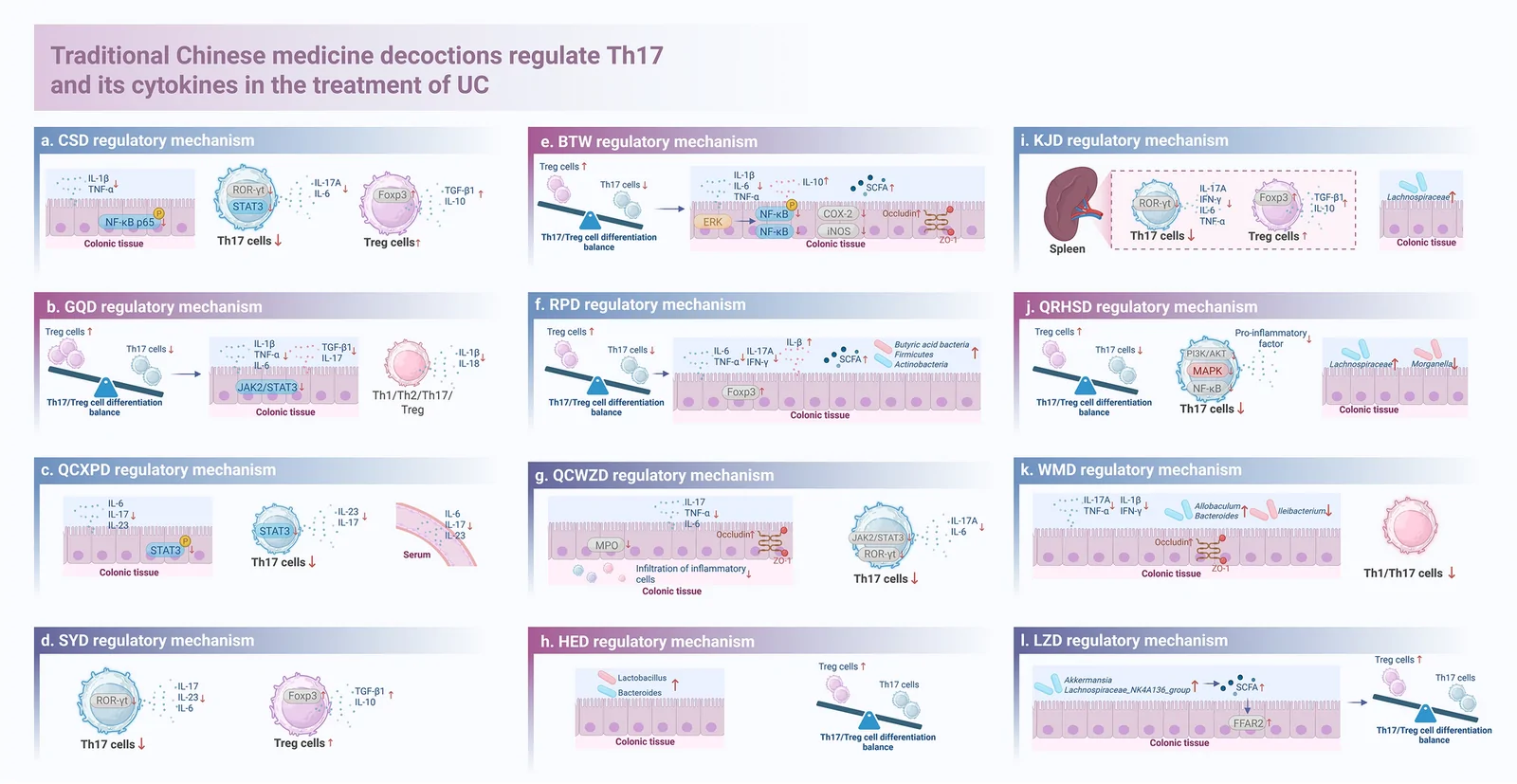
Interventional effect of TCM decoctions on Th17 related mechanism in UC

TCM formulations have numerous constituents. They are multi-purpose and treat various problems in inflammatory bowel disease. In recent years, more studies have investigated how these formulations control Th17 cells to maintain the immune imbalance in UC. Unlike single Chinese herbs, these formulations affect many signaling pathways. They can control Th17 cell differentiation, migration, and cytokine production. This contributes to immune regulation and mucosal barrier repair. This section will summarize the role of TCM formulations in regulating Th17 pathways in UC (Table 2, Fig. 5).

**Table 2:** Interventional effect of TCM decoction on Th17 related mechanism in UC

Name	Th17 related mechanism	Model	References
Compound Sophorae Decoction	Suppresses Th17 cell differentiation and inflammatory cytokine expression by downregulating AHR-mediated IL-6/STAT3/ROR-γt signaling and reducing IL-17A levels, while enhancing Treg responses via upregulating FOXP3, TGF-β1, and IL-10 to restore Th17/Treg balance	DSS Mice & TNBS Mice	[22, 146]
Gegen-Qinlian Decoction	Modulates Th17/Treg and Th1/Th2 immune balance by inhibiting IL-6/JAK2/STAT3 signaling and regulating inflammatory cytokine expression	DSS Mice and TNBS Mice	[21, 148]
QingChang-XiaoPi Decoction	Modulates pathogenic Th17 cells by inhibiting IL-6/IL-23/IL-17 axis and STAT3 phosphorylation level	DSS Mice	[149]
Shaoyao Decoction	Regulates Th17/Treg balance by inhibiting HIF-1α and STAT3 signaling, reducing IL-17 and RORγt expression, and enhancing FoxP3, IL-10, and TGF-β1 levels	TNBS Rat	[151]
Baitouweng Decoction	Suppressing Th17-related inflammation, enhancing intestinal barrier function, inhibiting ERK/NF-κB signaling, and promoting short-chain fatty acid production	DSS Mice	[153]
Rhubarb-Peony Decoction	Suppressing Th17 cell responses through modulation of gut microbiota and SCFA levels, downregulating IL-17A,IFN-γ and other pro-inflammatory cytokines to restore intestinal immune balance	DSS Mice	[155]
Qingchang Wenzhong Decoction	Suppresses Th17 cell differentiation by inhibiting the IL-6/JAK2/STAT3 signaling pathway, with reduced transcriptional activation of RORγt and IL-17A	DSS Mice	[157]
Huanglian-ejiao decoction	Correcting Th2/Th1 and Treg/Th17 immune imbalance, reducing inflammatory cytokines and transcription factors, and modulating gut microbiota by enriching beneficial bacteria and inhibiting opportunistic pathogens	TNBS Mice	[159]
Kuijie decoction	Suppresses Th17 cell differentiation and pro-inflammatory cytokine expression by modulating gut microbiota and related metabolites (glutamine and tryptophan), while enhancing Treg responses to restore Th17/Treg balance	DSS Mice	[161]
Qing-Re-Hua-Shi Decoction	Suppresses Th17-mediated inflammation by modulating gut microbiota and metabolites, and inhibiting PI3K/AKT, NF-κB, and MAPK pathways	DSS Mice	[162]
Wumei Decoction	Suppressing Th1/Th17 cell differentiation, downregulating proinflammatory cytokines such as IL-17A, and modulating gut microbiota composition	DSS Mice&Rag1^−/−^ mice	[164]
Lizhong Decoction	Mitigates Th17-mediated colitis by enriching SCFA-producing gut microbes and activating the FFAR2 pathway to suppress Th17 cell differentiation through a microbiota–metabolite–immunity axis	DSS Mice	[166]

UC: ulcerative colitis; Th17: T helper 17 cells; TCM: Traditional Chinese medicine; Treg: regulatory T cells; TGF-β: transforming growth factor-beta; IL-: interleukin-; STAT3: signaling and activator oftranscription protein 3; RORγt: retinoid-related orphan nuclear receptor gamma t; CXCL: chemokine ligand; G-CSF: granulocyte colony-stimulating factor; TNF-α: tumor necrosis factor-alpha; Foxp3: Forkhead-like protein 3; NF-κB: nuclear factor-kappa B; MAPK: mitogen-activated protein kinase; C/EBP: CCAAT/enhancer binding protein; MMP: mitochondrial membrane potential; TRAF6: TNF receptor associated factor 6; pTh17: pathogenic Th17 cells; IFN-γ: interferon-Gamma; RORC: receptor-associated orphan receptor; RegIIIγ: Regenerating islet-derived protein III-gamma; GM-CSF: granulocyte–macrophage colony-stimulating factor; AREG: Amphiregulin; non-pTh17: non-pathogenic Th17 cells; α-SMA: α-smooth muscle actin; ROS: reactive oxygen species; MUC2: mucin 2; TLR: Toll-like receptor; CRP: C-reactive protein; IBD: inflammatory bowel disease; eNAMPT: extracellular nicotinamide phosphoribosyl transferase; miR: micro RNA; CCR6: chemokine (C-C motif) receptor 6; DAI: disease activity index; MHCII: major histocompatibility complex class II; JAK2: Janus Kinase 2; ZO-1: zonula occludens-1; ROCK: rho-associated kinase; DSS: dextran sulphate sodium; AhR: aryl hydrocarbon receptor; SCFA: short-chain fatty acids; GPR: G-protein coupled receptor; CCM: curcumin-chitosan microspheres; PCR: polymerase chain reaction; IGF2BP1: Insulin-like growth factor 2 mRNA binding protein 1; LRP5: Low-density lipoprotein receptor-related protein 5; S1P2: sphingosine-1-phosphate receptor 2; Rho GTP: Rho GTPase; lncRNAlong: non-coding RNA; MIAT: myocardial infarction associated transcript; ILC1: innate lymphoid cells 1; CSD: Compound Sophorae Decoction; p-NF-κB: phosphorylation-nuclear factor kappa B; FICZ: Fluorescein isothiocyanate; GQD: Gegen-Qinlian Decoction; QCXPD: QingChang-XiaoPi Decoction; SYD: Shaoyao Decoction; HIF1-α: hypoxia-inducible factor-1 alpha; BTWD: Baitouweng Decoction; p-ERK: phosphorylation-extracellular regulated protein kinases; COX-2: cyclooxygenase-2; iNOS: inducible nitric oxide synthase; RPD: Rhubarb-Peony Decoction; QCWZD: Qingchang Wenzhong Decoction; MPO: myeloperoxidase; HED: Huanglian-ejiao Decoction; KJD: Kuijie Decoction; QRHSD: Qing-Re-Hua-Shi Decoction; PI3K/AKT: phosphatidylinositol 3-kinase/protein Kinase B; WMD: Wu Mei Decoction; LZD: Lizhong Decoction; FFAR2: free fatty acid receptor 2

#### Compound Sophorae Decoction

Compound Sophorae Decoction (CSD) is a classic Chinese herbal formula made of six herbs: Sophora flavescens root, Sanguisorba officinalis charcoal, Indigo naturalis, Bletilla striata rhizome, Panax notoginseng powder, and Glycyrrhiza uralensis root. It clears heat (i.e., downregulates pro-inflammatory cytokines), detoxifies (i.e., suppresses oxidative stress and inflammatory mediators), and regulates the intestines (i.e., restores Th17/Treg balance and improves mucosal barrier function). This formula has been produced based on the clinical experience of Professor Heng Fan from Wuhan Union Hospital in the treatment of active-phase UC [145]. Xu et al. explored how CSD affects the Th17/Treg balance. The results revealed that CSD ameliorates the symptoms and reduces colonic damage in mice. It downregulated inflammatory markers, like IL-1β, TNF-α, and p-NF-κB p65 in the colon. CSD also reduced the abundance of Th17 cells in the spleen and mesenteric lymph nodes. It lowered ROR-γt, IL-17A, IL-6, and STAT3 in colonic tissue and suppressed Th17 cell function. At the same time, CSD increased the abundance of Treg cells and upregulated FOXP3, TGF-β1, and IL-10, thereby alleviating the symptoms of UC [22]. Another study showed that CSD works through the AHR pathway, downregulating the AHR agonist FICZ in the colon, suppressing AHR-mediated IL-6/STAT3/RORγt pathway, and reducing the number of Th17 cells. It was also shown to upregulate FOXP3 levels, thereby promoting Treg cell development and suppressing inflammation [146].

#### Gegen-Qinlian Decoction

Gegen-Qinlian Decoction (GQD) originates from the Treatise on Febrile Diseases. GQD consists of four ingredients: Pueraria root (Ge Gen), Scutellaria root (Qin Lian), Coptis root (Huang Lian), and Licorice root (Gan Cao). It purges heat (i.e., downregulates pro-inflammatory cytokines, like IL-6, IL-17, and TNF-α), detoxifies (i.e., suppresses oxidative stress and pro-inflammatory mediators), raises yang, ameliorates diarrhea, restores intestinal barrier function, and balances the stomach and spleen (i.e., restores gut immune homeostasis). Recent studies have indicated that GQD possesses significant anti-inflammatory and immune-modulatory properties [147]. GQD suppresses the inflammatory and immune responses in rodent models of UC. Zhao et al. administered GQD (1.5 and 7.5 g/kg/day) orally to mice with DSS-triggered UC. They observed that GQD mitigated the manifestations of UC, downregulated myeloperoxidase and proinflammatory mediators, such as IL-1β, TNF-α, and IL-6, and lowered the concentrations of TGF-β1 and IL-17. This correlated with the suppression of the IL-6/JAK2/STAT3 signaling, restoring Th17/Treg balance, and decreasing immune cell infiltration into the colon [148]. Hu et al. delivered GQD rectally (2.96 and 11.83 g/kg/day) in a preclinical model of TNBS-induced inflammatory bowel disease. They noted that GQD normalized the Th2/Th1 and Treg/Th17 balance, decreased the abundance of Th2 and Th1 cells and their transcription factors GATA3 and T-bet, and modulated diverse immune-related cytokines, such as IL-2, IL-12, IL-5, IL-13, IL-6, IL-10, and IL-17A. GQD also reduced IL-1β and IL-18 concentrations, thereby alleviating UC [21].

#### QingChang-XiaoPi Decoction

QingChang-XiaoPi Decoction (QCXPD) is another formula used to treat UC. It contains several herbs, like Coptis rhizome, Hedyotis herb, Sanguisorba root, Atractylodes rhizome, Aquilaria wood, Paeonia root, and Hirudo. QCXPD clears heat (i.e., inhibits pro-inflammatory signaling), transforms dampness, promotes qi circulation, resolves stagnation, ameliorates diarrhea, and restores intestinal barrier function. Jia et al. discovered that QCXPD can alleviate the clinical manifestations of UC, such as diarrhea, abdominal cramps, distension. In a murine model of DSS-induced UC, QCXPD mitigated intestinal irritation. It decreased the plasma and tissue concentrations of pro-inflammatory mediators, including IL-6, IL-17, and IL-23. QCXPD also inhibited STAT3 signaling and diminished the abundance of pTh17. Mechanistically, it suppressed the IL-23/IL-17 cascade and STAT3 signaling, offering anti-inflammatory and immunomodulatory benefits [149].

#### Shaoyao Decoction

Shaoyao Decoction (SYD) is a well-known formula obtained from Suwen: Mechanisms of Disease and Principles of Preservation of Life. It is used to treat diarrhea, abdominal pain, and intestinal damp-heat. It contains peony root, angelica root, fried licorice root, Coptis rhizome, wood fragrant, rhubarb, and betel nut. It clears heat (i.e., downregulates pro-inflammatory cytokines, such as IL‑1β and IL‑6), transforms dampness (i.e., modulates the composition of gut microbiota, restores microbial metabolites, like short-chain fatty acids (SCFAs), and improves intestinal barrier and water balance), regulates qi (i.e., enhances intestinal motility and smooth muscle function), relieves pain (i.e., reduces inflammation-induced abdominal pain), and helps balance the intestines (i.e., restores Th17/Treg balance and strengthens the integrity of the mucosal barrier) [150]. SYD showed strong anti-inflammatory and immune-regulating effects in animal models of TNBS-induced UC, mainly by regulating Th17/Treg cell balance. SYD improved the symptoms of colitis and colon damage in rats, reduced disease activity and histopathological scores, lowered the proportion of Th17 cells, and downregulated their cytokines (IL-17, IL-23, IL-6, and RORγt). It also increased the abundance of Treg cells and downregulated IL-10, TGF-β1, and FoxP3. SYD minimized Th17 cell-mediated intestinal damage by lowering HIF-1α, STAT3, and p-STAT3 levels, restoring immune balance, and treating UC [151].

#### Baitouweng Decoction

Baitouweng Decoction (BTWD), derived from Zhang Zhongjing’s Treatise on Febrile Diseases during the Eastern Han Dynasty, comprises four components: Bai Tou Wu, Huang Lian, Huang Bai, and Qin Pi. It clears heat (i.e., downregulates pro-inflammatory cytokines, like IL-6 and IL-1β), detoxifies (i.e., reduces inflammatory mediators and attenuates oxidative stress), cools blood (i.e., suppresses NF-κB signaling and preserves the integrity of intestinal mucosa), alleviates diarrhea, and restores intestinal barrier function. It is conventionally utilized for managing damp-heat dysentery, diarrhea, and abdominal discomfort. Contemporary research suggests that BTWD possesses anti-inflammatory and antibacterial properties and protects the intestines [152]. Miao et al. reported that BTWD diminishes inflammation and maintains the integrity of the intestinal barrier in mice with DSS-induced UC. It lowered DAI scores, minimized colonic injury, downregulated IL-1β, IL-6, and TNF-α concentrations, and upregulated the anti-inflammatory factor IL-10. Additionally, it improved the expression of tight junction proteins (Occludin and ZO-1) in intestinal epithelial cells, inhibited p-NF-κB and p-ERK, and suppressed COX-2 and iNOS levels. Moreover, BTWD promoted the synthesis of short-chain fatty acids, such as acetate, propionate, isobutyrate, and isovalerate, in the gut. BTWD alleviated UC by blocking the ERK/NF-κB pathway, enhancing intestinal permeability, and harmonizing the Th17/Treg balance [153].

#### Rhubarb-Peony Decoction

Rhubarb-Peony Decoction (RPD), from the Essential Prescriptions of the Synopsis Golden Chamber, was originally used for treating early intestinal abscesses and damp-heat stagnation. It consists of five herbs: rhubarb, peony root bark, winter melon seeds, peach kernels, and mirabilite. RPD clears heat (i.e., downregulates pro-inflammatory cytokines, such as IL‑6, IL‑1β, and TNF‑α), disperses nodules (i.e., modulates local intestinal inflammation and immune cell infiltration), promotes blood flow (i.e., improves mucosal microcirculation and supports tissue repair), resolves stasis (i.e., enhances intestinal motility and local immune regulation), and helps unblock the bowel (i.e., restores intestinal transit and resolves obstruction) [154]. RPD significantly mitigated weight loss, intestinal atrophy, and cellular injury in mice with DSS-modelled UC. Mechanistically, it modulated intestinal microbiota and the Th17/Treg equilibrium. RPD elevated the abundance of beneficial microorganisms, such as *Clostridium butyricum*, *Firmicutes,* and *Actinobacteria*, and diminished the proportion of detrimental strains, like *Proteobacteria* and *Bacteroides*. Moreover, it enhanced the production of SCFAs. It also altered the Th17/Treg balance in mesenteric lymphoid tissues, reduced the expression levels of IL-6, TNF-α, IL-17A, and IFN-γ in the gut, and increased the expression of TGF-β and Foxp3, thereby restoring the immune homeostasis in the digestive tract [155].

#### Qingchang Wenzhong Decoction

Qingchang Wenzhong Decoction (QCWZD) represents an effective Chinese herbal formula for the treatment of UC. It includes Coptis rhizome, processed ginger, Sophora japonica flower, indigo powder, carbonized Panax notoginseng, honeysuckle flower, ginseng, and licorice. It has been clinically shown to help treat the symptoms of UC [156]. Studies have indicated that QCWZD acts via multiple targets to alleviate DSS-induced UC. Twenty-seven active components were identified in serum samples, laying the groundwork for further studies. Treatment with QCWZD reduced myeloperoxidase (MPO) activity and inflammatory cell infiltration in the colon. It also downregulated the serum levels of pro-inflammatory factors, like IL-6, TNF-α, and IL-17A, while increasing the expression of tight junction proteins Occludin and ZO-1, thereby improving intestinal barrier function. Additionally, QCWZD reduced the abundance of Th17 cells, blocked the IL-6/JAK2/STAT3 pathway, lowered the expression of RORγt and IL-17A, and inhibited Th17 cell differentiation [157].

#### Huanglian-ejiao Decoction

According to the Treatise on Febrile Diseases, Huanglian-ejiao Decoction (HED) consists of five herbs: Coptis rhizome, Scutellaria root, Paeonia root, chicken egg yolk, and donkey-hide gelatin. It cools the body, feeds the yin (i.e., restores mucosal integrity and the anti-inflammatory balance), lowers the fire (i.e., suppresses pyroptosis and downregulates ROS/IL‑1β), prevents bleeding, and balances the ying and wei qi (i.e., restores Th17/Treg immune homeostasis). It is used for inflammatory conditions, such as insomnia and bleeding [158]. Studies have indicated that HED exerts strong anti-inflammatory effects in mice with TNBS-induced UC. HED was found to adjust CD4^+^T cell differentiation, restore Th2/Th1 and Treg/Th17 balances, downregulate inflammatory cytokines, and improve intestinal barrier function. It also improved the survival rate of mice, maintained their body weight, and preserved the healthy colon tissue in mice with TNBS-induced UC. Additionally, HED modulated the composition of gut microbiota by increasing the abundance of beneficial microbiota, like *Lactobacillus* and *Bacteroides*, and suppressing the proportion of harmful pathogens, like *Helicobacter*. These findings support the ability of HED to suppress inflammation in UC by modulating the immune response and modifying the composition of the gut microbiome [159].

#### Kuijie Decoction

Kuijie Decoction (KJD) is a formula from Tongji Hospital in Wuhan. It treats UC and comprises eight herbs, including Herba Eupatorium, Portulaca oleracea, Bletilla striata, Sanguisorba officinalis, Sophora japonica flower, and fried Glycyrrhiza uralensis [160]. KJD improved clinical symptoms in the mouse model of DSS-induced UC. Specifically, it reduced the abundance of Th17 cells in the spleen and downregulated the expression of IL-6, IL-17A, IFN-γ, and TNF-α. It also hindered Th17 cell differentiation, increased the proportion of Treg cells, and upregulated Foxp3, IL-10, and TGF-β, restoring the balance between Th17 cells and Treg cells. It also increased the abundance of beneficial bacteria, like Lachnospiraceae, and upregulated glutamine and tryptophan [161].

#### Qing-Re-Hua-Shi Decoction

Qing-Re-Hua-Shi Decoction (QRHSD) includes eight herbs: dandelion, white peony root, Sanguisorba root, betel nut, Sophora flower, Bletilla root, red peony root, and Euryale seed. It suppresses inflammation, regulates the immune response, and modulates the composition of gut microbiota. In patients with UC, QRHSD offers therapeutic effects with few side effects. QRHSD was reported to decrease the abundance of Th17 cells and increase the number of Treg cells. RNA-seq and network pharmacology analysis revealed that QRHSD blocks NF-κB, MAPK, and PI3K/AKT pathways and downregulates pro-inflammatory factors. 16S rRNA and metabolomics revealed that it also alters the composition of gut microbiota by increasing the abundance of *Lactobacillus* and reducing the abundance of *Morganella*. QRHSD was also indicated to affect Th17 metabolism, including bile acid and amino acid levels [162].

#### Wumei Decoction

Wumei Decoction (WMD) originated from the "Jueyin Disease Chapter" in Treatise on Febrile Diseases and has evolved into a classic formula through refinements by physicians throughout the ages. It includes ten herbs: black plum, Coptis, dried ginger, Sichuan pepper, Phellodendron, Aconite, Cinnamon twig, Ginseng, Angelica, and Evodia. WMD offers anti-inflammatory and antitumor effects [163]. Wang et al. employed two mouse models to evaluate the therapeutic effects of WMD on UC and investigate its immunomodulatory mechanisms. One model involved the induction of chronic colitis via repeated stimulation with 3% DSS. The other model was an autoimmune colitis model established by transferring CD45RB^high^CD4^+^ T cells into Rag1^−/−^ immunodeficient mice, which highly reproduced T cell-mediated intestinal inflammation. In both models, WMD significantly alleviated weight loss, bloody stools, and colon shortening and reduced histopathological damage to the colonic tissue. Immunologically, WMD suppressed the excessive activation of intestinal CD4 + T cells, significantly reduced the Th1/Th17 cell ratio, and lowered the expression of pro-inflammatory factors (e.g., TNF-α, IL-1β, IFN-γ, and IL-17A). Therefore, it likely alleviated colitis by inhibiting the Th17-dominant immune-inflammatory pathway. Additionally, WMD upregulated tight junction proteins, such as Occludin, ZO-1, and E-cadherin, to enhance the integrity of the intestinal barrier. Moreover, WMD increased the abundance of *Allobaculum* and *Bacteroides* and decreased the abundance of *Ileibacterium* to restore the composition of gut microbiota [164].

#### Lizhong Decoction

Lizhong Decoction (LZD) is a well-known formula from Zhang Zhongjing's Treatise on Febrile Diseases in China's Han Dynasty. Ginseng, asarum, licorice, and white atractylodes are included in this formula. It warms the middle, dispels cold, raises Qi, and strengthens the Spleen [165]. Studies have shown that LZD regulates gut microbiota and metabolism. LZD was shown to prevent weight loss and intestinal damage in a mouse model of DSS-induced UC by modulating Th17 + and Treg + cells. Killing gut microbiota using antibiotics undermined the beneficial effects of LZD on colitis. 16S rDNA sequencing showed that LZD alters the composition of gut microbiota and promotes the growth of beneficial bacteria, such as *Akkermansia* and *Lachnospiraceae_NK4A136_group*, which produce SCFAs. These SCFAs activate FFAR2 receptors and regulate immune pathways to inhibit the development of many Th17 cells. Thus, LZD can be used to treat Th17-related inflammation [166].

### Common pathways and chemical patterns in TCM-mediated Th17 regulation

TCM extracts, monomers, and formulas have different origins and chemical compositions, but their effects on Th17-related inflammation are often mediated through several shared pathways. These pathways include IL-6/JAK2/STAT3/RORγt, AHR, NF-κB/MAPK, and gut microbiota-related metabolism. Recent reviews have shown that natural products and TCM formulas regulate UC through linked targets, such as inflammatory signaling, immune balance, gut barrier repair, and intestinal microbiota regulation [167].

IL-6 activates JAK2/STAT3 signaling, thereby enhancing RORγt expression and promoting Th17 cell differentiation. NF-κB and MAPK upregulate the production of inflammatory cytokines, which can further strengthen Th17-related inflammation. AHR is linked to Th17/Treg balance, IL-22 production, epithelial repair, and mucosal immune balance. Gut microbiota and microbial metabolites can also affect these pathways by changing the local intestinal environment.

Flavonoid-rich compounds show some common features. Kurarinone and baicalein are often linked to JAK2/STAT3, AHR, NF-κB, oxidative stress, and epithelial barrier regulation. These effects may be attributed to the polyphenolic structure of flavonoids. This structure may help flavonoids affect redox-related and ligand-responsive signaling.

Alkaloids show wider immune and metabolic effects. Matrine, oxymatrine, and berberine have been linked to JAK/STAT signaling, inflammatory cytokine production, intestinal barrier repair, inflammasome activity, and gut microbiota remodeling. These changes may affect Th17/Treg balance.

Compound formulas are different from isolated monomers. Decoctions contain many chemical groups, such as flavonoids, alkaloids, saponins, polysaccharides, organic acids, and other small molecules. Their effects may originate from several components [168]. Compound Sophorae Decoction, Gegen-Qinlian Decoction, Baitouweng Decoction, Qingchang Wenzhong Decoction, and Lizhong Decoction all affect Th17/Treg balance, inflammatory cytokine release, mucosal barrier integrity, and gut microbiota composition. In these formulas, some components may inhibit IL-6/JAK2/STAT3/RORγt-driven Th17 differentiation, some components may regulate AHR/IL-22-mediated epithelial repair, and others may change microbial metabolites, such as SCFAs and tryptophan derivatives.

The link between chemical structure and pathway selection is still unclear. Most studies used whole extracts or full formulas. Few studies compared purified compounds with different structures in the same model. Dosage, absorption, intestinal metabolism, microbial transformation, and herb-herb interactions may also change the final effect. Future studies should conduct chemical fingerprinting, pharmacokinetic analysis, target validation, and structure–activity analysis [169].

## Summary and Outlook

This review discussed how TCMs regulate Th17 cells and their cytokines in UC. Th17 cells, as an important subset of CD4⁺ T-cells, play a central role in UC. TCM monomers, such as paeoniflorin, kurarinone, astragaloside IV, baicalein, curcumin, cinnamaldehyde, and salidroside, can modulate Th17-related immune responses by regulating inflammatory pathways, cytokine production, and gut microbiota. Some formulas of TCM, including Compound Sophorae Decoction, Gegen-Qinlian Decoction, QingChang-XiaoPi Decoction, Shaoyao Decoction, Kuijie Decoction, Baitouweng Decoction, etc., were also shown to regulate the Th17/Treg balance, thereby reducing inflammatory injury and protecting the intestinal mucosal barrier.

Most mechanistic studies have been conducted in mouse and rat models of DSS- or TNBS-induced colitis. They are suitable for studying epithelial injury and acute inflammation. However, they are not entirely representative of all people. Human UC is a chronic, relapsing, and heterogeneous disease. Genetics, gut microbiota, environmental factors, diet, and previous treatments all affect UC. Some clinical studies and randomized controlled trials have been published regarding the use of TCMs in UC. Recently, more attention has been paid to randomized, double-blind, placebo-controlled trials in this field. However, for the particular Th17-modulating formulas mentioned in this review, direct human RCT data on Th17/Treg-related immune endpoints are still lacking. Most of the clinical trials have only focused on symptoms, clinical response, recurrence, and mucosal healing. Furthermore, immune markers, such as Th17 cells, Treg cells, IL-17, IL-23, RORγt, Foxp3, and other mucosal biomarkers, were not consistently measured. In the future, multi-center randomized controlled trials with consistent outcome measures are needed to assess clinical remission, endoscopic healing, histological improvement, relapse prevention, safety, quality of life, and Th17/Treg-related immune biomarkers.

Mechanistic studies are still rare. Many studies have reported changes in the Th17/Treg ratio, cytokine secretion, signaling pathways, and gut microbiota composition; however, few have determined the causal association between particular TCM compounds or microbial metabolites and Th17 cell behavior or mucosal repair. In the future, we will employ an integrated approach and combine microbiomics, metabolomics, transcriptomics, proteomics, single-cell sequencing, fecal microbiota transplantation, and immune cell transfer to clarify how individual compounds or formulations affect Th17 differentiation, plasticity, pathogenicity, and tissue localization. Functional validation in germ-free or antibiotic-treated models and pathway inhibition can also be conducted.

There are many TCM formulas, and there is no standardization. Different origins of raw materials, processing, extraction, and dosage are likely to result in different results. In the future, chemical fingerprinting and quantitative component analysis will be conducted to determine the main components responsible for Th17-related immune regulation through batch consistency tests, pharmacokinetic evaluations, and identification of active ingredients. UC is a heterogeneous disease, and Th17-related immune features vary with disease stage, inflammation severity, microbiota profile, prior treatments, and patient characteristics. Future studies on the use of TCM for UC should investigate biomarker-guided individualized therapy. The potential combination of TCM with conventional treatment, such as 5-ASA, corticosteroids, immunosuppressants, biologics, small-molecule drugs, probiotics, or dietary interventions, should be evaluated to determine whether TCM can improve efficacy, reduce relapse, or limit adverse effects.

## Data Availability

Data availability is not applicable to this article as no new data were created or analyzed in this study.

## Abbreviations

5-ASA: 5-aminosalicylic acid
ACT1: NF-κB activator 1
AhR: aryl hydrocarbon receptor
ALKBH5: AlkB homolog 5, RNA demethylase
AREG: amphiregulin
BACH2: BTB domain and CNC homolog 2
BATF: basic leucine zipper ATF-like transcription factor
BTWD: Baitouweng decoction
C/EBP: CCAAT/enhancer-binding protein
C10orf99: chromosome 10 open reading frame 99
CCM: curcumin–chitosan microspheres
CCR6: C-C motif chemokine receptor 6
CD4: cluster of differentiation 4
CD8: cluster of differentiation 8
CD86: cluster of differentiation 86
COX-2: cyclooxygenase-2
CRP: C-reactive protein
CSD: Compound Sophorae decoction
CXCL: C-X-C motif chemokine ligand
DAI: disease activity index
DSS: dextran sulfate sodium
EGFR: epidermal growth factor receptor
eNAMPT: extracellular nicotinamide phosphoribosyltransferase
ERK: extracellular signal-regulated kinase
FFAR2: free fatty acid receptor 2
FICZ: 6-formylindolo[3,2-b]carbazole
Foxp3: forkhead box P3
G-CSF: granulocyte colony-stimulating factor
GM-CSF: granulocyte-macrophage colony-stimulating factor
GPR: G protein-coupled receptor
GPR15: G protein-coupled receptor 15
GPR15L: G protein-coupled receptor 15 ligand
GQD: Gegen-Qinlian decoction
HED: Huanglian-Ejiao decoction
HIF-1α: hypoxia-inducible factor-1α
HRas: GTPase H-Ras
IBD: inflammatory bowel disease
IFN-γ: interferon-γ
IGF2BP1: insulin-like growth factor 2 mRNA-binding protein 1
IL: interleukin
IL-17RA: interleukin-17 receptor A
IL-17RC: interleukin-17 receptor C
IL-23R: interleukin-23 receptor
ILC1: group 1 innate lymphoid cells
ILC3: group 3 innate lymphoid cells
iNOS: inducible nitric oxide synthase
JAK: Janus kinase
JAK2: Janus kinase 2
KJD: Kuijie decoction
lncRNA: long non-coding RNA
LRP5: low-density lipoprotein receptor-related protein 5
LZD: Lizhong decoction
m6A: N6-methyladenosine
MAPK: mitogen-activated protein kinase
MHCII: major histocompatibility complex class II
MIAT: myocardial infarction associated transcript
miR: microRNA
MMP: matrix metalloproteinase
MPO: myeloperoxidase
mTOR: mechanistic target of rapamycin
MUC2: mucin 2
NF-κB: nuclear factor-κB
non-pTh17: non-pathogenic Th17 cells
PCR: polymerase chain reaction
p-ERK: phosphorylated extracellular signal-regulated kinase
PGAM: phosphoglycerate mutase
PI3K/AKT: phosphatidylinositol 3-kinase/protein kinase B
p-NF-κB: phosphorylated nuclear factor-κB
p-STAT3: phosphorylated signal transducer and activator of transcription 3
pTh17: pathogenic Th17 cells
QCWZD: Qingchang Wenzhong decoction
QCXPD: QingChang-XiaoPi decoction
QRHSD: Qing-Re-Hua-Shi decoction
RCT: randomized controlled trial
RegIIIγ: regenerating islet-derived protein IIIγ
RhoA: Ras homolog family member A
Rho-GTP: GTP-bound Rho
ROCK: Rho-associated coiled-coil-containing protein kinase
RORC: RAR-related orphan receptor C
RORγt: retinoic acid-related orphan receptor γt
ROS: reactive oxygen species
RPD: Rhubarb-Peony decoction
S1P2: sphingosine-1-phosphate receptor 2
SCFAs: short-chain fatty acids
SPTLC1: serine palmitoyltransferase long chain base subunit 1
STAT: signal transducer and activator of transcription
STAT3: signal transducer and activator of transcription 3
SYD: Shaoyao decoction
TCM: traditional Chinese medicine
TGF-β: transforming growth factor-β
Th1: T helper 1 cells
Th2: T helper 2 cells
Th17: T helper 17 cells
TLR: Toll-like receptor
TNBS: 2,4,6-trinitrobenzenesulfonic acid
TNF-α: tumor necrosis factor-α
TRAF6: TNF receptor-associated factor 6
Treg: regulatory T cells
UC: ulcerative colitis
WMD: Wumei decoction
ZO-1: zonula occludens-1
α-SMA: α-smooth muscle actin
